# CD5阳性弥漫大B细胞淋巴瘤诊断和治疗中国专家共识（2026年版）

**DOI:** 10.3760/cma.j.cn121090-20251218-00599

**Published:** 2026-03

**Authors:** 中华医学会血液学分会淋巴细胞疾病学组 中国抗癌协会淋巴瘤专业委员会

## Abstract

CD5阳性弥漫大B细胞淋巴瘤（CD5^+^ DLBCL）是弥漫大B细胞淋巴瘤中少见的免疫亚型，常表现为高度侵袭性、疾病进展快、预后较差。在以利妥昔单抗为基础的免疫化疗时代，该类患者生存获益仍有限。近年来，随着基因分型等精准评估手段的发展及个体化免疫靶向治疗研究的推进，对CD5⁺DLBCL的认识不断深入，部分患者的治疗结局有望改善。为进一步规范诊疗实践，中华医学会血液学分会淋巴细胞疾病学组及中国抗癌协会淋巴瘤专业委员会组织相关专家制定本共识，用于指导中国CD5⁺DLBCL患者的标准化诊断与治疗。

## Abstract (en)

CD5-positive diffuse large B-cell lymphoma（CD5⁺ DLBCL）is a rare immunophenotypic subtype of DLBCL, typically characterized by high aggressiveness, rapid disease progression, and poor prognosis. Even in the era of rituximab-based immunochemotherapy, survival benefits for these patients remain limited. In recent years, with the development of precision assessment tools such as genotyping and advances in research on personalized immunotargeted therapies, the understanding of CD5⁺ DLBCL has deepened, offering the prospect of improved treatment outcomes for some patients. To further standardize diagnosis and treatment practices, the Lymphoid Disease Group, Chinese Society of Hematology, Chinese Medical Association and the Lymphoma Expert Committee of China Anti-Cancer Association have organized relevant experts to formulate this consensus, aiming to guide the standardized diagnosis and treatment of CD5⁺ DLBCL patients in China.

CD5阳性弥漫大B细胞淋巴瘤（CD5-positive diffuse large B cell lymphoma，CD5^+^DLBCL）是弥漫大B细胞淋巴瘤（DLBCL）中少见的免疫学亚型，临床进展迅速，预后较差。在以利妥昔单抗为基础的免疫化疗时代，CD5⁺DLBCL患者的生存获益仍然有限。近年来，随着基因分型等精准评估方法的临床应用，以及个体化免疫靶向治疗的相关临床研究进展，对CD5⁺DLBCL的认识也不断深入，部分患者的近期疗效和远期生存有望获得改善。为进一步规范中国CD5^+^DLBCL患者的诊断与治疗实践，中华医学会血液学分会淋巴细胞疾病学组和中国抗癌协会淋巴瘤专业委员会结合国际临床研究前沿及国内多中心临床研究工作，组织相关领域专家制定本共识，用于指导中国CD5⁺DLBCL患者的规范化诊断和治疗。

一、定义

CD5^+^DLBCL是一种少见的DLBCL亚型，肿瘤细胞异常表达CD5分子。CD5是T细胞的生物标志物，但也可表达于某些肿瘤性B细胞，如慢性淋巴细胞白血病（chronic lymphocytic leukemia，CLL）/小B细胞淋巴瘤（small B-cell lymphoma，SLL）、套细胞淋巴瘤（mantle cell lymphoma，MCL）及部分成熟B细胞（如B1-a细胞）[1]。在2008年版造血与淋巴组织肿瘤WHO分类中，CD5⁺DLBCL被列为一种独立的淋巴瘤免疫学亚型。CD5^+^DLBCL在全部DLBCL中占5％～17％，多见于老年患者，临床表现为高度侵袭性，分期较晚，骨髓、中枢神经系统（central nervous system，CNS）及其他结外器官受累比例较高[2]–[3]。

二、诊断、鉴别诊断、分期和预后

（一）诊断

1. 临床特征：CD5⁺DLBCL患者发病的中位年龄约为65岁，女性略多于男性[4]–[6]。60％～70％患者在确诊时已处于疾病晚期（Ann Arbor分期Ⅲ/Ⅳ期），骨髓受累发生率约为20％，CNS受累发生率约为30％[7]。结外侵犯较为常见，常见部位包括皮肤、肾上腺和乳腺等。血清乳酸脱氢酶（LDH）水平常升高[8]，诊断时国际预后指数（International Prognostic Index，IPI）评分普遍较高[9]–[10]。

2. 病理诊断：

（1）病理检查：推荐淋巴结完整切除或结外病灶的充分组织活检，尽量避免采用细针穿刺活检，尤其应避免选择明显炎症反应区域的淋巴结作为取材部位。手术过程中应避免组织挤压，以确保病理质量。若为胃肠道病灶，建议在超声内镜引导下进行活检。CNS受累者可在磁共振成像（MRI）评估后通过立体定向穿刺方式获取组织样本。对于深部病灶，建议在计算机体层成像（CT）或超声引导下穿刺活检。若仅存在CNS受累，患者体质状态或病灶位置特殊，难以获得组织病理学诊断，可考虑行脑脊液形态、流式细胞术、细胞因子如IL-10、IL-6水平测定等进一步明确诊断。另外，骨髓活检或腹壁脂肪盲检也是可疑血管内大B细胞淋巴瘤患者可以考虑的获取病理诊断的方法。获取的组织应尽快固定保存，具备条件时建议同步采用流式细胞术进行免疫表型分析。术前行PET-CT检查有助于指导取材部位的选择，提高活检的阳性率和准确性。

（2）形态学：CD5⁺DLBCL表现为淋巴结结构弥漫性破坏，多数病例以中心母细胞样形态为主，少数可见免疫母细胞型，亦有罕见的间变型报道。组织学常见星空现象、坏死、溃疡及核分裂象，间质纤维化较为少见。总体而言，与CD5^-^DLBCL相比，形态学特征尚缺乏特异性。

（3）免疫组织化学（immunohistochemistry，IHC）评估：IHC是诊断CD5^+^ DLBCL的重要依据。CD5^+^ DLBCL常表现为非生发中心B细胞样（non-germinal center B-cell like，non-GCB）表型[11]，CD10^−/+^，BCL-6^+^/MUM1^+^/BCL-2^+^，可见MYC/BCL2双表达，不表达Cyclin D1。推荐的IHC标志物组合至少包括CD20、PAX5、CD3、CD5、CD43、CD10、BCL-2、BCL-6、MUM1、C-MYC、CD30、Cyclin D1、CD21、CD23、LEF1及EBER原位杂交检测。除CD20外，CD19和CD22亦为潜在的嵌合抗原受体T细胞（chimeric antigen receptor T cell，CAR-T细胞）治疗靶点，建议同步完善检测。CD5阳性判读标准为：肿瘤细胞表达Pan-B标志（如CD20、PAX5或CD19等）的同时，CD5表达阳性（阳性细胞比例>20％）。在判读CD5表达时，应结合Pan-B及Pan-T标志（如CD3、CD2、CD7）进行比对，以明确CD5的表达存在于肿瘤性B细胞而非背景T细胞上。即使为弱阳性表达，只要肿瘤性大B细胞中CD5表达超过20％，亦可视为CD5阳性。

（4）流式细胞术：推荐采用两步法进行淋巴组织的流式细胞术免疫分型分析。初筛管建议包含以下抗体：CD19、CD20、CD5、CD10、膜轻链κ、膜轻链λ、CD38和CD56。CD5⁺DLBCL通常在流式细胞术图形中呈现FSC/SSC较大的细胞群，并表现出膜轻链限制性表达。然而，亦存在部分病例膜轻链阴性，此时应进一步检测胞质轻链κ/λ，以排除幼稚B细胞肿瘤，并判断是否存在轻链限制。在免疫表型方面，CD5⁺DLBCL需与CLL Richter转化（Richter transformation，RT）、MCL及边缘区淋巴瘤（marginal zone lymphoma，MZL）等鉴别。但由于仅依靠流式细胞术免疫分型难以准确区分上述疾病，因此需结合患者的临床病史、组织病理学及分子检测结果，综合判断以明确诊断。

（5）分子病理：建议对CD5⁺DLBCL病例开展FISH检测以评估MYC、BCL-2、BCL-6等基因重排情况，并结合二代测序（next-generation sequencing，NGS）等分子检测手段明确遗传学异常，用于辅助鉴别诊断、指导分子分型及靶向治疗。部分CD5⁺DLBCL病例可见MYC或BCL-6基因重排，但通常缺少BCL-2重排。考虑到MYC和BCL-6重排常涉及非免疫球蛋白（non-IG）伙伴基因，推荐采用断裂探针进行FISH检测。而BCL-2重排多与IG基因相关，宜采用融合探针检测。对于形态学及免疫表型提示存在基因重排但断裂探针检测结果阴性的病例，建议进一步进行融合探针FISH或NGS检测，以获取更全面的遗传信息，用于辅助诊断及风险分层。石峰等[12]的研究纳入18例原发CD5⁺DLBCL患者，88.9％表达BCL-2蛋白，72.2％表达C-MYC，C-MYC/BCL-2双阳性率为66.7％；MYD88 L265P与CD79B Y196F突变发生率均为16.7％，PD-1与PD-L1在部分肿瘤及免疫细胞中亦表达阳性，提示该类患者可能从联合免疫治疗中获益。在NGS分析中，CD5⁺DLBCL较CD5^-^ DLBCL更常出现PIM1、MYD88、CD79B、KMT2D等基因突变及CDKN2A失活性变异（包括无义突变和拷贝数缺失），而DUSP2与TET2突变则较少见[13]。

（6）遗传特征：比较基因组杂交（comparative genomic hybridization，CGH）分析表明，在CD5⁺DLBCL和CD5^-^DLBCL中，均有超过20％的病例存在基因组异常，常见的染色体获得区域包括1q21-q31、1q32、3p25-q29、5p13、6p21-p25、7p22-q31、8q24、11q23-q24、12q13-q21、16p13、18和X获得；常见的缺失区域包括1p36、3p14、6q14-q25、6q27、9p21和17p11-p13[14]。尽管CD5⁺与CD5^-^DLBCL的总体基因组改变模式存在一定相似性，但在CD5⁺DLBCL中，10p14-p15和19q13的获得以及1q43-q44和8p23的缺失更具特征性[15]–[16]。将其与MCL、CLL及Richter综合征的CGH谱系进行对比，可见CD5⁺DLBCL具有明显不同的遗传学特征。Yoshioka等[17]进一步报道，原发性CD5⁺DLBCL患者中8p21和11q13染色体异常发生率较高，其主要断点集中于8p21、11q13和3q27。其中，携带8p21的患者常呈现更具侵袭性的临床表现，如疾病状态为晚期、LDH水平升高、功能状态评分差，预后更差[17]。

（二）鉴别诊断

1. CLL/SLL的RT：RT是CLL/SLL从惰性向侵袭性进展的临床现象，其中以转化为DLBCL最为常见[18]，总体转化率为2％～10％。RT的临床表现通常包括不明原因的发热、体重减轻、淋巴结快速增大、LDH水平显著升高及结外浸润引起的多器官功能障碍等[18]–[19]。与RT相关的高危因素包括高龄、淋巴结较大（>3 cm）、IGHV突变阴性、del（17p）、TP53突变及NOTCH1突变等[20]–[21]。

需要注意的是，单纯出现增殖中心扩大或Ki-67增高并不足以诊断DLBCL型RT，仍需满足DLBCL弥漫性大细胞浸润、结构破坏等病理诊断标准。RT相关的大B细胞淋巴瘤在形态上与原发性DLBCL高度相似，鉴别时需结合完整临床病史及是否存在CLL/SLL残留病灶等因素综合判断。RT相关DLBCL常伴TP53和NOTCH1突变，亦可见CDKN2A缺失及MYC易位。虽然MYC异常在RT中相对常见，具有一定鉴别诊断价值，但CDKN2A缺失等基因改变亦可出现在CD5⁺DLBCL中，因此其特异性较差[22]。此外，免疫球蛋白基因重排的克隆性分析有助于判断RT来源于CLL/SLL同源克隆还是新发的非同源性克隆性B细胞淋巴瘤。

2. MCL：MCL主要见于老年男性，常累及结外部位，尤其是胃肠道和咽部淋巴环。母细胞型或多形性MCL亦可表达CD5，其肿瘤细胞通常呈中到大，形态上易与CD5⁺DLBCL或高级别B细胞淋巴瘤混淆，但可通过Cyclin D1和SOX11的表达区分。尽管少数DLBCL亦可表达Cyclin D1，但通常不伴随CD5表达。因此，若具有大B细胞形态的淋巴瘤同时表达CD5和Cyclin D1，应首先考虑多形性MCL的可能，FISH或NGS检测若提示CCND1易位，可进一步支持MCL诊断。Cyclin D1的核内强阳性为MCL相对特异性的免疫表型，经典型MCL常同时表达SOX-11，且95％以上患者可检测到特征性染色体易位t（11;14）（q13;q32），均有助于鉴别。需要注意的是，部分多形性MCL可不表达Cyclin D1，亦无CCND1重排，更易与CD5⁺DLBCL混淆。此时，建议补充SOX11免疫染色及CCND2/CCND3重排检测，以排除相应变异导致的MCL；必要时可行NGS检测以获取更全面的遗传信息。其他MCL中常见的突变，如ATM、TP53、CDKN2A，亦可在CD5⁺ DLBCL中检测到，鉴别诊断的价值较低。

3. 其他CD5^+^的B细胞淋巴瘤：除CD5⁺DLBCL外，其他表达CD5的B细胞淋巴瘤亚型还包括少见的MZL、滤泡性淋巴瘤、血管内大B细胞淋巴瘤等[23]。尽管这些亚型的部分病例也可出现CD5阳性，但通常具有相对特异的形态学、免疫组化及分子遗传学特征，有助于与CD5⁺DLBCL鉴别。

（三）分期和预后

1. 分期：分期依据淋巴结及结外受累范围进行评估，推荐采用2014年修订的Lugano分期标准（表1）[24]。此外，可进一步根据有无B症状［不明原因发热（>38 °C，连续3 d）；或夜间盗汗；或6个月内不明原因的体重下降>10％］对患者进行分组，A组：无B症状；B组：有B症状。

**表1:** 2014版淋巴瘤Lugano分期

分期	侵犯范围
局限期	
Ⅰ期	仅侵及单一淋巴结区域（Ⅰ期），或侵及单一结外器官不伴淋巴结受累（ⅠE期）
Ⅱ期	侵及横膈一侧≥2个淋巴结区域（Ⅱ期），可伴同侧淋巴结引流区域的局限性结外器官受累（ⅡE期）
Ⅱ期伴大包块	纵隔包块MMR>0.33，其他部位最大直径≥10 cm
进展期	
Ⅲ期	侵及横膈肌上下淋巴结区域，或横膈以上淋巴结区受累伴脾脏受累（ⅢS期）
Ⅳ期	侵及淋巴结引流区域外的结外器官

**注** MMR：肿块最大径/胸腔最大径

2. 预后：在免疫化疗时代，临床采用改良的IPI（NCCN-IPI）（表2）[25]对CD5⁺DLBCL患者进行预后风险分层。对于老年患者，可参考年龄调整的IPI（aa-IPI）评分系统（表2）。此外，中国淮海淋巴瘤工作组（HHLWG）开展的一项多中心研究纳入207例中国CD5⁺DLBCL患者，结果显示，以血清白蛋白水平与外周血淋巴细胞计数构成的预后营养指数（PNI）是影响总生存（OS）的独立预后因素，提示营养与免疫状态可能在该亚型患者的预后中发挥重要作用[26]。福建医科大学附属肿瘤医院的一项研究纳入29例CD5⁺DLBCL患者，显示Ann Arbor分期晚期、病理类型为non-GCB、未接受利妥昔单抗治疗显著缩短患者的OS期[27]。

**表2:** NCCN-IPI和aa-IPI模型的危险因素及分值

预后模型	因素	分值（分）
NCCN-IPI	年龄	
	>40岁且≤60岁	1
	>60岁且<75岁	2
	≥75岁	3
	LDH	
	ULN<LDH≤ULN×3	1
	>ULN×3	2
	Ann Arbor分期（Ⅲ/Ⅳ期）	1
	ECOG PS≥2分	1
aa-IPI	Ann Arbor分期（Ⅲ/Ⅳ期）	1
	LDH>ULN	1
	ECOG PS≥2分	1

**注** NCCN-IPI：改良的国际预后指数；aa-IPI：年龄调整的国际预后指数；LDH：乳酸脱氢酶；ECOG PS：美国东部肿瘤协作组体能状态评分；ULN：正常值上限

三、治疗

（一）治疗前评估

1. 病史询问和体格检查：全面采集患者的既往史及本次发病相关信息，包括有无B症状，血液、消化、神经、呼吸、心血管等系统的临床表现。体格检查应详尽评估浅表淋巴结、肝脾及其他可能发生结外受累的脏器，特别需重视乳腺、睾丸和CNS等结外部位的体格检查，同时完善美国东部肿瘤协作组体能状态评分（ECOG PS）等。

2. 实验室检查：包括血常规、肝肾功能、血糖、病毒感染指标（乙型肝炎病毒、丙型肝炎病毒、人类免疫缺陷病毒、EB病毒）、免疫球蛋白水平、淋巴细胞亚群分析、血清蛋白电泳、β_2_-微球蛋白、LDH、细胞因子等；根据临床情况，可进一步检测心肌酶、肌钙蛋白、脑钠肽等心功能相关指标。疑似CNS受累患者，应行脑脊液常规、生化及细胞因子如IL-10等检测。

3. 主要脏器功能：包括心脏彩超、心电图、肺功能检查、肝肾功能评估等；必要时行脑电图了解CNS功能状态。

4. 骨髓检查：建议行骨髓细胞学、骨髓活检，必要时行骨髓流式细胞术检查，明确骨髓受累情况。

5. CNS评估：对于CNS受累风险高的患者，建议行头颅MRI检查，并完善脑脊液常规、生化及流式细胞术检测等。

6. 超声/影像学检查：建议进行浅表淋巴结、腹部脏器、腹膜后淋巴结等部位彩超检查；同时推荐胸部CT、心脏彩超、头颅MRI、全腹增强CT、PET-CT等影像学检查，以明确病变范围与脏器功能状态。

7. 生殖功能评估：对于有生育需求的育龄期患者，应进行性激素水平检测，并在必要时行卵子或精子冻存，提前做好生殖保护计划。

8. 心理评估和健康宣教：可采用心理健康评估量表对患者进行初步心理状态筛查，必要时提供心理支持与干预。应对患者进行疾病宣教，帮助其合理应对治疗过程中的身心挑战。

（二）治疗方案

1. 一线治疗策略与探索方向：以利妥昔单抗为基础的免疫化疗方案在CD5⁺DLBCL患者中整体疗效有限，尤其是在non‑GCB亚型患者中，未能显著改善患者生存，推荐积极开展临床研究。一项纳入102例CD5⁺DLBCL患者的研究显示，虽然利妥昔单抗治疗可以提高患者的4年无进展生存（PFS）率，但并未使OS率获益[28]。此外，利妥昔单抗的应用也未能有效降低CD5⁺DLBCL患者CNS受累的风险，增加治疗强度的R-EPOCH方案（利妥昔单抗+依托泊苷+泼尼松+长春新碱+环磷酰胺+阿霉素）同样未带来额外的生存获益[29]，不推荐将强化化疗作为CD5⁺DLBCL患者的一线选择。联合甲氨蝶呤（methotrexate，MTX）的免疫化疗方案旨在控制CNS受累，患者的客观缓解率和OS获得改善[30]。中国台湾的一项研究纳入174例老年DLBCL患者，多因素分析表明，CD5阳性和IPI评分≥3分是患者预后不良的独立影响因素[31]。总体上，建议采用基于基因分型的精细化评估和个体化治疗策略，以提高治疗效果[32]–[33]。

HHLWG的一项多中心研究提示，中国CD5⁺DLBCL患者中54％为MCD分子亚型（即同时存在MYD88 L265P与CD79B突变的DLBCL亚型），相关生信分析也预测了基于现有分子靶向、表观遗传等药物联合治疗的可行性，未来值得探索[13]。此外，布鲁顿酪氨酸激酶抑制剂（BTKi）联合R-CHOP方案（利妥昔单抗+环磷酰胺+阿霉素+长春新碱+泼尼松）推荐用于CD5^+^DLBCL的一线治疗，以改善患者的近期疗效和长期生存率。上海交通大学医学院附属瑞金医院的研究进一步显示，CD5阳性non-GCB亚型DLBCL存在明显的脂质代谢异常，伴随CD36的高表达，可能促进免疫逃逸。在此基础上，该团队正在开展拓展性临床研究，尝试通过BTKi联合二甲双胍治疗逆转其不良预后，值得进一步验证。

Polarix研究已证实维泊妥珠单抗联合免疫治疗方案在高危DLBCL患者中已显示出一定的生存优势[34]，在特定条件下也可在CD5⁺DLBCL患者中进行前瞻性探索。在维持治疗方面，建议结合患者的临床分期、细胞起源及分子亚型等因素进行综合决策。基于CD5⁺DLBCL特有的分子生物学特征，BTKi在维持治疗中具有一定潜力，但仍需进一步开展前瞻性研究证实。对于遗传/功能性高危人群，亦可通过在一线治疗中开展CAR-T细胞治疗的临床研究进行前瞻性探索。

2. CNS受累的防治：CD5⁺DLBCL具有较高的CNS受累倾向，累计发生率为15％～33％[29],[35]。相关研究表明，该特性可能与部分神经系统相关功能基因的异常表达密切相关。联合利妥昔单抗的免疫化疗方案可提高CD5⁺DLBCL患者的完全缓解率，但并不能显著降低CNS受累的发生率[36]。一旦发生CNS受累，患者的OS期明显缩短，提示CNS受累与不良预后密切相关[37]–[38]。因此，建立从评估、预防到干预治疗的全程管理策略至关重要。临床上推荐常规开展头颅MRI与脑脊液相关检查，排查CNS高危或潜在受累患者，并根据风险等级制定预防性治疗方案。对于明确存在CNS受累及免疫豁免器官受累的患者，尤其强调一线针对CNS淋巴瘤治疗和CNS预防的重要性。联合CNS预防治疗策略能够有效改善患者的生存，一项日本的前瞻性临床研究采用R-EPOCH方案联合大剂量MTX治疗CD5⁺DLBCL，显著延长患者4年PFS期与OS期[39]。针对CD5⁺DLBCL患者CNS受累的防治，推荐综合应用鞘内注射、大剂量MTX、BTKi等方案进行强化治疗。以塞替派为基础的预处理方案，推荐用于适合自体造血干细胞移植（auto-HSCT）的患者。

3. 靶向药物的应用进展与前景：CD5⁺DLBCL具有独特的临床病理和分子遗传特征，其遗传背景还常伴有染色体不稳定性[40]。BTKi已被证实对MCD、non-GCB、双表达亚型及CD79B单突变患者均具有良好疗效，因此BTKi联合R-CHOP方案在CD5⁺DLBCL的治疗中具有重要价值。此外，CD5⁺DLBCL的基因特征与ABC亚型DLBCL具有高度相似性，Ma等[13]的研究通过突变谱分析提示，CD5⁺DLBCL中PIM1、KMT2D、BCL2等基因的突变频率显著升高。靶向治疗是未来值得探索的新方向，除BTKi外，PIM1抑制剂、组蛋白去乙酰化酶抑制剂及BCL-2抑制剂等新型靶向药物均具有潜在治疗价值[41]。CD5⁺DLBCL同样具有异质性，建议常规开展遗传与分子分型检测，以指导基于新型靶向药物的个体化治疗。

4. 造血干细胞移植的临床应用：在免疫和靶向治疗快速发展的背景下，auto-HSCT在DLBCL治疗中的地位面临挑战。对于CD5⁺DLBCL患者，auto-HSCT用于一线巩固治疗尚缺乏高质量的临床证据，亟需前瞻性临床研究验证。目前，对于年轻高危患者，前期诱导治疗获得良好疗效后仍可考虑进行auto-HSCT作为巩固治疗。推荐在临床研究的框架下探索联合新型药物、移植后维持治疗等多种策略，以进一步改善患者的预后。对于难治复发患者，移植的地位仍然重要，但挽救性auto-HSCT或异基因造血干细胞移植（allo-HSCT）并不能改善患者的生存[28]。推荐在造血干细胞移植前进行积极有效的再诱导治疗（二线化疗，免疫靶向及CAR-T细胞治疗等），争取改善患者移植前状态。造血干细胞动员推荐参照《淋巴瘤自体造血干细胞动员和采集中国专家共识（2020年版）》[42]制定造血干细胞采集策略，必要时可于中期疗效评估阶段进行造血干细胞动员及冻存。auto-HSCT的预处理方案以BEAM方案（卡莫司汀+依托泊苷+阿糖胞苷+美法仑）为主，若存在CNS受累，可考虑以塞替派为基础的强化清髓方案[43]。

5. 难治/复发患者的治疗策略：对于难治或复发CD5⁺DLBCL患者，强烈建议进行二次活检，以完善病理诊断及分子分型，尤其应包括基因测序以明确潜在的基因突变特征。同时，应重视CNS受累与结外病变的评估，为后续制定个体化治疗方案提供依据。治疗可参考B细胞淋巴瘤NCCN（2025.V3）指南推荐的二线免疫化疗方案，并探索联合多种新型药物，包括Pola、PD-1抑制剂、XPO1抑制剂、抗体药物偶联物（antibody-drug conjugate，ADC）及双特异性抗体（bispecific antibody，BsAb）等。对于初始治疗过程中未应用BTKi的患者，推荐在再次诱导治疗中积极纳入BTKi，以提高治疗应答率。Alinari等[28]采用auto-HSCT、allo-HSCT或联合移植治疗28例难治复发CD5⁺DLBCL，其中20例移植后复发。从首次造血干细胞移植至复发或因任何原因死亡的中位时间为4.9个月，提示造血干细胞移植仍不能改善CD5⁺DLBCL患者的生存。

CAR-T细胞治疗已被批准用于二线及以上难治复发DLBCL，并在多项研究中显示出良好疗效，5年OS率可达到42.6％，推荐作为难治复发CD5^+^DLBCL患者的治疗选择[44]。除经典的CD19靶点外，CD20、CD22等靶点的CAR-T细胞治疗也在开展临床研究，推荐患者积极参与相关临床试验。对于身体状况、器官功能良好的患者，可考虑接受CAR-T细胞治疗联合auto-HSCT。对于体能状况较差或存在部分器官功能障碍的患者，可考虑CAR-T细胞联合微移植，或先行CAR-T细胞治疗改善疾病状态，后续再行auto-HSCT，并可根据情况选择是否联合第二次CAR-T细胞治疗。部分不具备自体CAR-T细胞治疗条件的患者，可以考虑通用型CAR-T、CAR-NK细胞治疗等临床研究。部分体能状况较好，自体CAR-T细胞治疗无效或反复复发的患者可以在严格评估的前提下选择allo-HSCT联合供者CAR-T细胞治疗的临床研究。总体而言，应充分重视CAR-T细胞治疗在难治复发CD5^+^ DLBCL患者整体治疗体系中的关键作用，并考虑其与造血干细胞移植联合的策略。建议在患者身体状况允许的前提下，尽早采集并冻存外周血单个核细胞和造血干细胞。

6. 超高龄及虚弱患者的个体化管理：超高龄及体质虚弱CD5^+^DLBCL患者的临床特点包括治疗耐受性和依从性较差、脏器储备功能减退、对化疗敏感性下降，且常伴有复杂的分子和遗传特征。其治疗策略不应强调传统化疗的核心地位，而应以个体化和精细化管理为主导。建议80岁以上的超高龄患者及各类虚弱患者常规进行包括体能状态、心肺等主要脏器功能在内的全面评估，同时结合分子病理、基因突变及遗传特征的检测结果制定精准的治疗方案，并针对性选择适用的靶向药物。在初始治疗阶段，可优先考虑弱化化疗方案如R-mini-CHOP方案（利妥昔单抗+环磷酰胺+阿霉素+长春新碱+泼尼松），或采用免疫靶向主导的化疗减免方案，如ZR2方案（利妥昔单抗+来那度胺）等。对于进入难治复发阶段的超高龄或虚弱患者，亦可探索包括Pola-R-CHP方案（维泊妥珠单抗+利妥昔单抗+环磷酰胺+阿霉素+泼尼松）、PD-1抑制剂、XPO1抑制剂、ADC及BsAb等新型靶向免疫药物的治疗策略。上述方法在一定程度上可降低治疗相关不良反应，改善患者生活质量，并为不耐受常规方案的特殊患者提供更多治疗选择。

CD5^+^ DLBCL的治疗流程见图1。

**图1:**
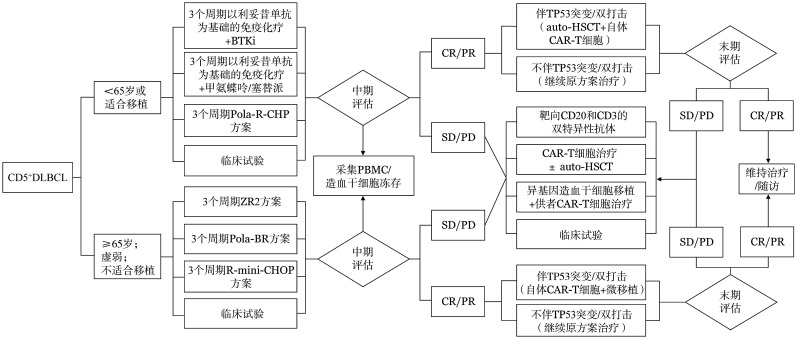
CD5阳性弥漫大B细胞淋巴瘤（CD5^+^DLBCL）的治疗流程 **注** BTKi：布鲁顿酪氨酸激酶抑制剂；Pola-R-CHP：维泊妥珠单抗+利妥昔单抗+环磷酰胺+阿霉素+泼尼松；ZR2：利妥昔单抗+来那度胺；Pola-BR：利妥昔单抗+维泊妥珠单抗+苯达莫司汀；R-mini-CHOP：利妥昔单抗+环磷酰胺+阿霉素+长春新碱+泼尼松；PBMC：外周血单个核细胞；CR：完全缓解；PR：部分缓解；SD：疾病稳定；PD：疾病进展；auto-HSCT：自体造血干细胞移植；CAR-T细胞：嵌合抗原受体T细胞

四、疗效评估标准

参照2014版Lugano标准（表3），优先建议行PET-CT及全身增强CT评估。

**表3:** 2014版Lugano疗效评价标准

疗效	部位	PET-CT（代谢缓解）	CT（影像学缓解）
完全缓解	淋巴结和结外病灶	Deauville评分法“1～3分”伴或不伴残余肿块	靶病灶（淋巴结）LDi≤1.5 cm且无淋巴结外病灶
	不可测病灶	不适用	消失
	器官肿大	不适用	恢复正常
	新发病灶	无	无
	骨髓	无骨髓FDG亲和性病灶证据	形态正常，如不能明确需流式细胞术检查阴性
部分缓解	淋巴结和结外病灶	Deauville评分法“4或5分”，伴摄取较基线降低，残余病灶可为任意大小	最多6个靶病灶SPD降低≥50％，如病灶过小，CT无法测量，5 mm×5 mm为默认值，不可见病灶为0 mm×0 mm
	不可测病灶	不适用	消失/正常，残余病灶未增大
	器官肿大	不适用	脾脏垂直径缩小值>原垂直径增大值的50％
	新发病灶	无	无
	骨髓	残留摄取高于正常骨髓组织但较基线低。如淋巴结缓解情况下骨髓持续存在结节性异常改变，需MRI或活检进一步诊断	不适用
无缓解或疾病稳定	淋巴结和结外病灶	Deauville评分法“4或5分”，代谢与基线相比无明显改变	最多6个靶淋巴结SPD降低<50％，且不符合疾病进展所有条件
	不可测病灶	不适用	没有与进展相符的增加
	器官肿大	不适用	没有与进展相符的增加
	新发病灶	无	无
	骨髓	与基线相比无变化	不适用
进展性疾病	单独的靶病灶（淋巴结/结节性肿块、结外病灶）	Deauville评分法“4或5分”摄取较基线增加，和（或）与中期或治疗结束评估一致的新发摄取增高	至少一个靶病灶进展即可诊断，淋巴结/结外病灶需同时符合下述要求：①LDi>1.5 cm；②PPD增加≥50％（较最小状态）；③LDi或SDi较最小状态增加0.5 cm（≤2 cm病灶）或1 cm（>2 cm病灶）
	器官肿大	不适用	脾大情况下，脾脏垂直径增大>原垂直径增大值的50％；若基线无脾大，垂直径需在基线基础上至少增加2 cm；新发或复发脾脏肿大
	不可测病灶	无	新发或不可测量病灶明确进展
	新发病灶	出现淋巴瘤相关的新发高代谢病灶（排除感染、炎症等），若未明确性质需进行活检或中期评估	新发淋巴结任意径线>1.5 cm；新发结外病灶任意径线>1 cm，如<1 cm，必须能够证实与淋巴瘤相关；明确与淋巴瘤相关的任意大小的病灶
	骨髓	新发或复发的高代谢摄取	新病灶或复发病灶

**注** FDG：氟代脱氧葡萄糖；LDi：病灶最长径；SPD：多个病灶的PPD之和；SDi：垂直于LDi的病灶最短径；PPD：LDi与SDi的乘积

五、随访

应在患者治疗过程中及治疗后关键随访节点系统开展疗效评估、不良事件监测及相关生物标志物检测。推荐定期评估临床缓解状态、药物相关不良反应、生化指标、免疫功能变化及EB病毒DNA等指标，结合影像学及实验室检查综合判断疾病进展及复发风险，重视继发肿瘤的监测与评估。推荐于患病2年内每3个月随访1次，2～5年每6个月随访1次，5年后每年随访1次。建议规范留取血液、骨髓及组织等多类型生物样本，以支持后续免疫监测及机制研究，推动样本库建设和精准医疗的发展。
